# *In situ* detection of alkaline phosphatase in a cisplatin-induced acute kidney injury model with a fluorescent/photoacoustic bimodal molecular probe

**DOI:** 10.3389/fbioe.2022.1068533

**Published:** 2022-11-23

**Authors:** Xingwang Chen, Zhiyang Yuwen, Yixing Zhao, Haixia Li, Kang Chen, Hongwen Liu

**Affiliations:** ^1^ Department of Hepatobiliary Surgery, The First Affiliated Hospital of Hunan Normal University (Hunan Provincial People’s Hospital), Hunan Normal University, Changsha, China; ^2^ Key Laboratory of Chemical Biology and Traditional Chinese Medicine Research, College of Chemistry and Chemical Engineering, Hunan Normal University, Changsha, China

**Keywords:** oxidative stress, acute kidney injury, alkaline phosphatase, fluorescent probe, photoacoustic (optoacoustic) imaging

## Abstract

Kidneys play an important part in drug metabolism and excretion. High local concentration of drugs or drug allergies often cause acute kidney injury (AKI). Identification of effective biomarkers of initial stage AKI and constructing activable molecular probes with excellent detection properties for early evaluation of AKI are necessary, yet remain significant challenges. Alkaline phosphatase (ALP), a key hydrolyzing protease, exists in the epithelial cells of the kidney and is discharged into the urine following kidney injury. However, no studies have revealed its level in drug-induced AKI. Existing ALP fluorescent molecular probes are not suitable for testing and imaging of ALP in the AKI model. Drug-induced AKI is accompanied by oxidative stress, and many studies have indicated that a large increase in reactive oxygen species (ROS) occur in the AKI model. Thus, the probe used for imaging of AKI must be chemically stable in the presence of ROS. However, most existing near-infrared fluorescent (NIRF) ALP probes are not stable in the presence of ROS in the AKI model. Hence, we built a chemically stable molecular sensor (CS-ALP) to map ALP level in cisplatin-induced AKI. This novel probe is not destroyed by ROS generated in the AKI model, thus allowing high-fidelity imaging. In the presence of ALP, the CS-ALP probe generates a new absorbance peak at 685 nm and a fluorescent emission peak at 716 nm that could be used to “turn on” photoacoustic (PA) and NIRF imaging of ALP in AKI. Levels of CS-ALP build up rapidly in the kidney, and CS-ALP has been successfully applied in NIRF/PA bimodal *in vivo* imaging. Through the NIRF/PA bimodal imaging results, we demonstrate that upregulated expression of ALP occurs in the early stages of AKI and continues with injury progression.

## Introduction

Drug-induced acute kidney injury (AKI) leads to serious morbidity and mortality (Blatt and Liebman, 2013). In the AKI process, drugs, such as cisplatin, first induce oxidative stress, which is followed by lysosomal damage and apoptosis. A recent survey indicated that AKI often continues unabated (Hsu et al., 2013). Unfortunately, the therapeutic schedule for AKI has not been highly effective due to lack of efficient biomarkers for early diagnosis and incomplete understanding of AKI pathogenesis (Charlton et al., 2014). Current clinical testing generally depends on the detection of urea nitrogen and serum creatinine in serum samples, which are often untimely, insensitive, and an indirect indicator of AKI (Charlton et al., 2014). In recent years, there has been extensive research focused on identification and verification of effective biomarkers for AKI. Previous studies have listed both potential and validated biomarkers of AKI, including small molecules, reactive oxygen species (O_2_^•−^, ONOO^−^, and ClO^−^), proteins, and enzymes (Liu H.-W. et al., 2020; Liu L. et al., 2020; Cheng et al., 2020; Huang and Pu, 2021). Among them, several enzymatic biomarkers, including N-acetyl-β-glucosaminidase (NAG), alanine aminopeptidase (AAP), and alkaline phosphatase (ALP), exist in the epithelial cells of the kidney and are discharged into the urine following kidney damage (Charlton et al., 2014). Additionally, fluorescent sensors have been developed for testing NAG, gamma glutamyl transferase (GGT), and AAP in AKI, and the results have demonstrated the overexpression of these enzymes in the early stages of AKI (Lv et al., 2018; Huang et al., 2019; Huang et al., 2020; Tan et al., 2021). However, the expression level of ALP in AKI has not been adequately assessed. Therefore, there is a need for construction of an activable molecular probe for detection of ALP in AKI to improve early detection of AKI.

**SCHEME 1:**
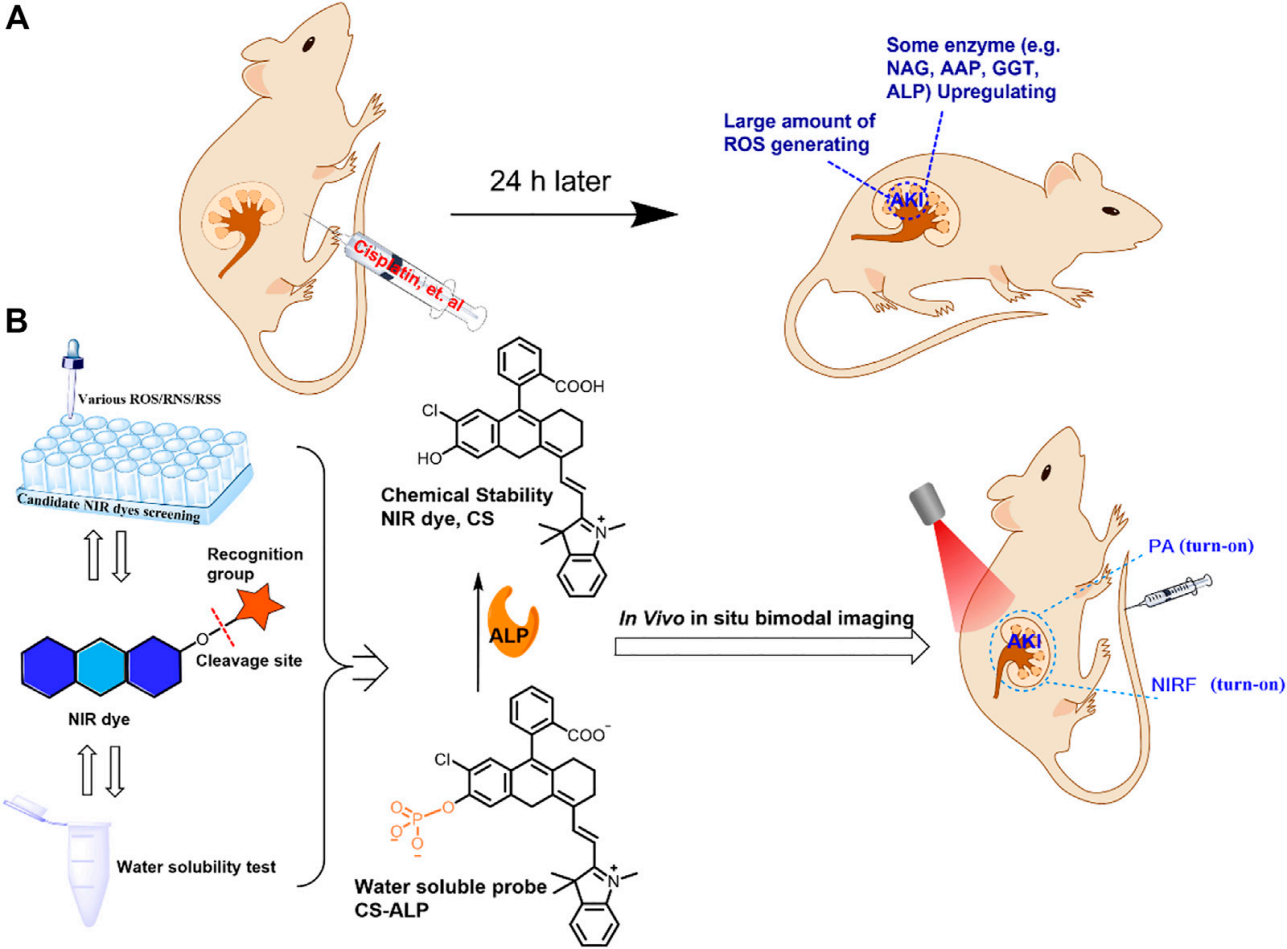
**(A)** Proposed reasons for choosing chemically stable dyes for constructing imaging probes for analysis of the drug-induced AKI mouse model. **(B)** Selection of a chemically stable NIR dye to construct a water-soluble probe, the structure of the proposed CS-ALP probe, and the mechanism of the reaction between CS-ALP and ALP and its application for *in vivo* NIRF/PA bimodal imaging.

ALP catalyzes the hydrolytic reaction and transphosphorylation of substrates. It is widely expressed, at low levels, in human tissues, including bone, liver, and kidney. However, its activity is remarkably increased in several human diseases (Orimo, 2010). Despite abundant research on ALP fluorescent probes (Supplementary Table S1, ESI) (Li et al., 2012; Zhou et al., 2016; Li et al., 2017; Liu et al., 2017; Liu et al., 2018; Zhang P. et al., 2019; Zhang Q. et al., 2019; Gao et al., 2019; Wang et al., 2021b), its role in AKI remains unclear due to the lack of a kidney-specific molecular probe. Furthermore, existing ALP fluorescent molecular probes are not suitable for testing and imaging of ALP in the AKI model. As summarized in Supplementary Table S1, previously developed near-infrared fluorescent (NIRF) ALP probes were based on cyanine dye, which is not stable in the presence of ROS in the AKI model; many studies have indicated that there is a large increase in ROS in AKI (Scheme 1A) (Jia et al., 2016; Cheng et al., 2019; Cheng et al., 2021; Li et al., 2021). In addition, the existing probes are hydrophobic and metabolized by the liver; therefore, no probe enrichment can occur in the kidney (Huang et al., 2017; Chen et al., 2021; Du et al., 2021; Yang et al., 2021). Development of a novel ALP fluorescent probe that is water soluble, chemically stable, and useful for *in situ* monitoring of ALP activity in the AKI model remains a significant challenge.

Herein, we report construction of a new NIRF probe, a chemically stable molecular sensor (CS-ALP), for studying AKI through measurement of ALP. CS-ALP was designed based on intramolecular charge transfer (ICT) and is composed of the chemically stable (CS) hemicyanine dye with a NIRF reporter and monophosphate linked to the hydroxyl group of the CS dye as an ALP cleavage site (Scheme 1B). Preliminary experiments and previous work have indicated that CS dye is stable in the presence of ROS at physiological concentration and shows mild water-solubility (Lv et al., 2018); thus, we chose CS dye for use in construction of an activable probe for high-fidelity imaging of ALP in AKI. In the presence of increasing concentrations of ALP, the monophosphate moiety is gradually hydrolyzed, resulting in gradual increase of the absorption maxima at 685 nm, which can be used for photoacoustic (PA) imaging. At the same time, the fluorescent emission peak at 716 nm was gradually enhanced, generating turn-on NIRF imaging. The probe displayed rapid response to ALP, high sensitivity (limit of detection: 0.26 U/L cal), and excellent specificity. Moreover, CS-ALP was successfully used for NIRF/PA bimodal imaging of ALP generated in cisplatin-stimulated human renal cortex proximal tubule epithelial cells (HK-2). Furthermore, using CS-ALP, the NIRF/PA bimodal *in vivo* visualization of ALP level in cisplatin-induced AKI was achieved for the first time, with results demonstrating that upregulated expression of ALP occurs at the early stages of AKI and continues with AKI progression.

## Experimental design

General reagents and all required materials, instruments, the probe synthesis protocol, and experimental data are provided in the Supporting Information (ESI).

### Spectrophotometric experiments

The probe was dissolved in DMSO to produce a stock solution (1.0 × 10^−4^ M). To obtain good optical performance of the probe, we used 5% DMSO as co-solvent. Since phosphate could inhibit the activity of ALP, we used 10 mM tris-buffered saline (TBS, pH 8.0) for *in vitro* experiments. Both the UV-Vis absorption and fluorescent spectrum experiments were carried out in 10 mM TBS (pH 8.0). The fluorescent emission spectra were tested using a Hitachi F7000 spectrophotometer, with the excitation wavelength at 680 nm and the emission wavelength ranging from 700 to 900 nm. The test solutions were kept at 37°C for 30 min and then analyzed using spectrophotometry.

### Fluorescence imaging in live cells

Cells were co-incubated with the probe in 10 mM TBS (pH 7.4). Cytotoxicity was assessed using the MTS assay. Cells were seeded in a 25-mm glass-bottom dish and grown for 24 h to approximately 85% confluency, followed by the live-cell confocal imaging experiments, for which the cells were divided into three groups as follows. The first group included HeLa cells that were incubated with 5 μM CS-ALP for 30 min. The cells were then washed with TBS buffer before imaging. In the second group, after pretreatment with Na_3_VO_4_ for 4 h, HeLa cells were incubated with 5 μM CS-ALP for 0.5 h and then washed with TBS prior to imaging. The third group included control cells without CS-ALP. For the cisplatin-induced nephrotoxicity model in HK-2 cells, the experiment was divided into four groups. The first group included HK-2 cells that were treated with 5 μM CS-ALP. The second and third groups included HK-2 cells that were incubated with cisplatin, 500 μM or 1000 μM, for 4 h, respectively, and then co-incubated with 5 μM CS-ALP for 0.5 h. The fourth group included cells that were pre-incubated with 50 μM Na_3_VO_4_ in the presence of cisplatin (1 mM) for 4 h and then treated with 5 μM CS-ALP for 0.5 h. Confocal fluorescence imaging of cells was performed using an Olympus FV1000 laser confocal microscope (Japan).

### Cisplatin-induced acute kidney injury

The drug-induced AKI mouse model was constructed following methods detailed in our previous report. Liu et al. (2020a) cisplatin was dissolved in 0.9% saline. BALB/c mice were intraperitoneally preinjected with cisplatin (20 mg/kg) for 24 h or 48 h. Live cells and live mouse experiments were in agreement with institutional animal care and use regulations, according to protocol No. SYXK (Xiang) 2022-0007, approved by Laboratory Animal Center of Hunan.

#### Detection of ALP in urine and blood samples collected from the cisplatin-induced AKI mouse model

Six BALB/c mice, with average initial body weight of 20 g, were fed in a metabolic cage after intraperitoneal preinjection of cisplatin (20 mg/kg), with urine samples collected at 24 h and 48 h after injection. Blood samples were drawn *via* the retro-orbital sinus at 24 h and 48 h after intraperitoneal administration of cisplatin. the samples were then tested immediately by adding 50 or 100 μL of the urine or blood sample to the test solution (total volume 1 ml) followed by recording of the fluorescence emission spectrum. The urine and blood of heathy mice were also tested as controls.

#### Determination of ALP in urine and blood samples using the commercially available ALP probe 4-methylumbelliferyl phosphate (4-MUP)

The 4-MUP probe was purchased from Sigma. The ALP level in urine and blood samples was then determined by measuring the fluorescence emission peak at 450 nm with *λ*_ex_ at 360 nm using a 4-MUP probe.

#### *In vivo* imaging of ALP in the cisplatin-induced AKI mouse

After intraperitoneal administration of cisplatin (20 mg/kg) for 24 h or 48 h, the CS-ALP probe (0.15 mg/kg in 20% DMSO/saline solution) was intravenously injected. The probe could not be completely solubilized in water at such a high concentration, so DMSO was added to improve solubility. Next, *in vivo* NIRF and PA imaging were carried out.

## Results and discussion

### Optimized design and synthesis of CS-ALP

**FIGURE 1:**
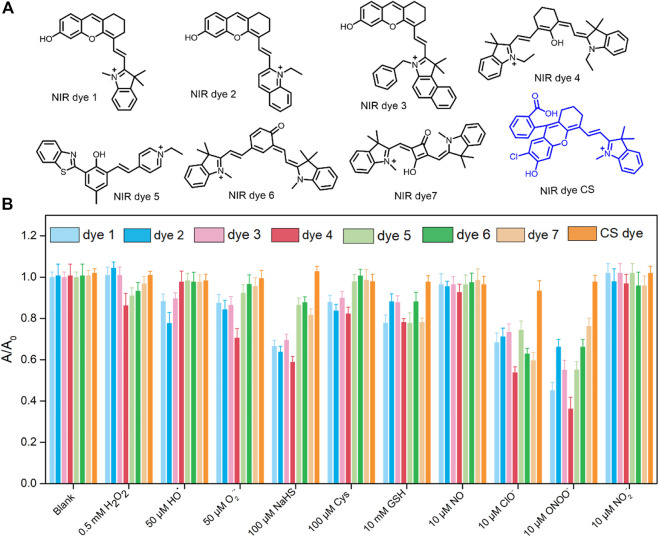
**(A)** Structure of hydroxyl containing NIR dyes 1–7 and the CS dye; dyes 1–5 were used for the constructed NIR ALP fluorescent probes. **(B)** Absorption intensities of the aforementioned dyes (5 μM) compared to various agents in an aqueous solution (PBS/EtOH = 8/2, pH 7.4). The absorption intensity of free dye (5 µM) was defined as 1.0. The tested solution was kept at room temperature for 0.5 h before analysis and data collection.

Drug-induced AKI is accompanied by oxidative stress. Many studies have indicated that high levels of ROS are generated by cells under oxidative stress (Mao et al., 2017; Mao et al., 2022). Thus, the probe used for imaging of AKI must be chemically stable in the presence of ROS. As proof of concept, we first studied the reactivity of the CS dye with typical oxidants and nucleophiles, including H_2_S, Cys, GSH, H_2_O_2_, O_2_^•−^, •OH, ONOO^−^, and ClO^−^. As exhibited in Figure 1, the absorption intensity of CS at 685 nm remained unchanged with the addition of test species at physiological concentrations. In contrast, seven other kinds of NIR dyes, five of which (dyes 1–5) have been used for constructing NIR ALP fluorescent probes (Li et al., 2012; Li et al., 2017; Zhang P. et al., 2019; Zhang Q. et al., 2019; Gao et al., 2019; Wang et al., 2021b), were also tested. The results indicated that dyes 1–7 were not stable in the presence of O_2_^•−^, •OH, ONOO^−^, and ClO^−^. These ROS have been shown to increase in concentration during drug-induced AKI (Lv et al., 2018; Huang et al., 2020). The results demonstrated that the existing NIRF ALP probes are not suitable for *in vivo* imaging and measurement of ALP levels in the drug-induced AKI model. In addition, for AKI *in vivo* imaging, probes must be water soluble at low concentration to allow enrichment in the kidney. We generated the probe through phosphorylation of the hydroxyl group of the CS fluorophore. The log P values of the probe and CS dye were 2.238 and 3.644, respectively, under pH 8.0 buffer conditions; as a control, the log P value of rhodamine B, a well know water-soluble dye, was calculated to be 2.032 under the experimental condition. The log P values indicated that CS-ALP has good water solubility and the potential for use in imaging to assess renal metabolism. To our knowledge, CS-ALP is the first molecular probe suitable for NIRF/PA bimodal *in vivo* detection of ALP in drug-induced AKI. The synthesis methodology for CS-ALP is shown in Supplementary Scheme S1, with structures assessed by ^1^H NMR and MS (ESI).

### Spectral response of the probe to ALP

**FIGURE 2:**
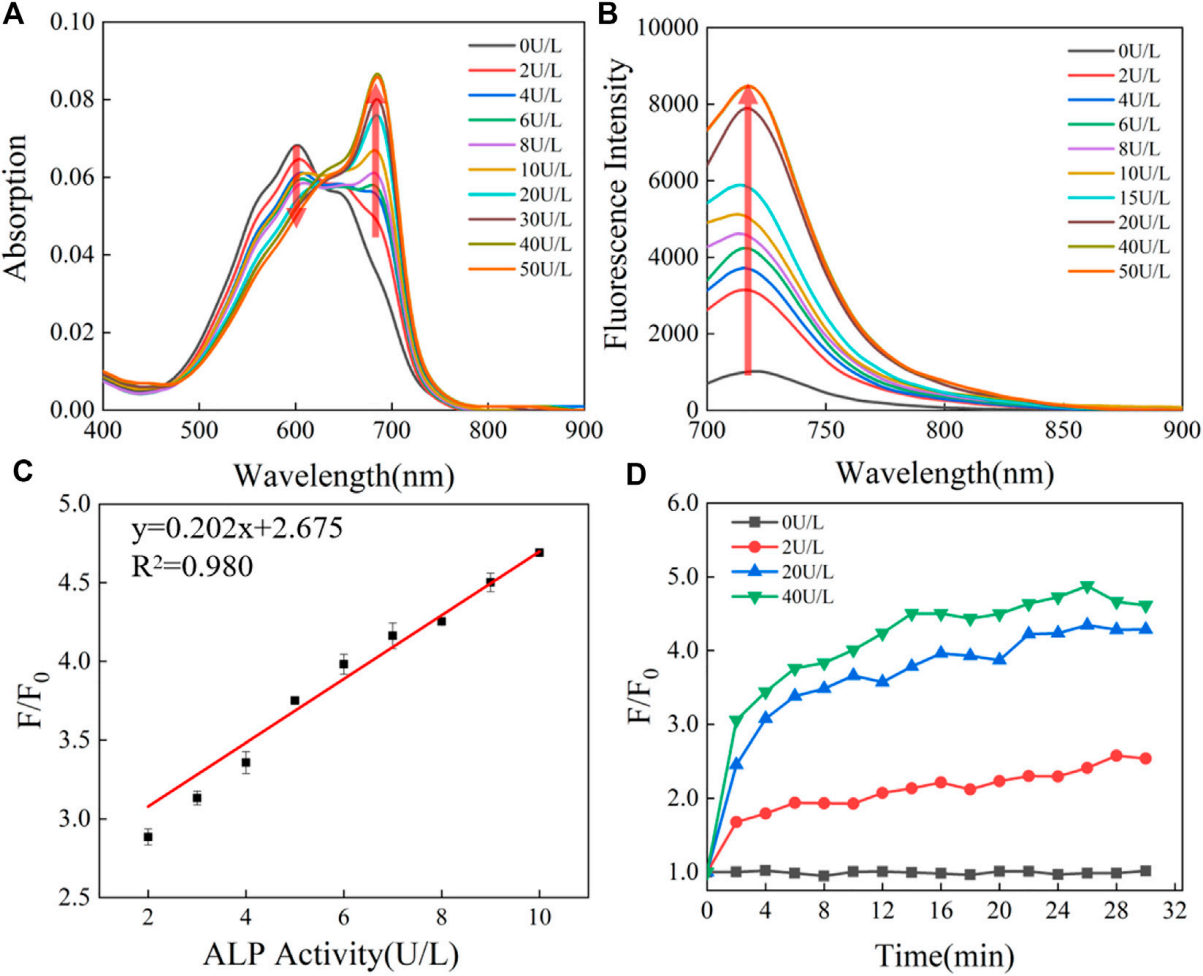
**(A)** Absorption and **(B)** emission spectra of 5 μM CS-ALP in the presence of different concentrations of ALP. **(C)** Calibration curves. **(D)** Fluorescence intensity at 716 nm *vs*. the reaction time with varying levels of ALP activity. The tests were carried out in aqueous solution (5% DMSO TBS buffer solution, pH 8.0) after incubation at 37°C for 0.5 h. *λ*_ex_ = 680 nm.

After synthesizing the probe, we conducted a spectral study of the reaction between CS-ALP and ALP. CS-ALP showed a maximum absorption peak at 600 nm. After addition of ALP, a new absorption peak appeared at 685 nm. The increased absorption intensity displayed a good linear relationship with the activity of ALP at 0–10 U/L (Figure 2A, and Supplementary Figure S1, ESI). Concomitantly, the fluorescence emission peak at 716 nm grew dramatically upon increased ALP activity (Figure 2B). According to the fluorescence titration experiment, the fluorescence intensity at 716 nm exhibited a good linear relationship with ALP at 0–10 U/L (Figure 2C), with a calculated detection limit of 0.26 U/L. The catalytic activity of the probe in response to ALP was then assessed. Figure 2D depicts the fluorescence dynamics of the reaction of the probe to ALP at different concentrations (0, 2, 20, and 40 U/L). The results indicate that high-activity ALP allows the probe to crack quickly, resulting in a dramatic increase in fluorescence intensity. The fluorescence intensity of the reaction system is essentially balanced at about 26 min. At the same time, in the absence of ALP, there was no significant change in the fluorescence intensity of the reaction system (Supplementary Figure S2, ESI), and the photo-stability of the probe and the CS dye is good (Supplementary Figure S3). Together, these results indicate that CS-ALP is stable at physiological pH and could be applied in bioimaging.

To further demonstrate the specificity of the reaction between CS-ALP and the enzyme, the specificity of the probe for various potentially biologically relevant substances was assessed. As shown in Supplementary Figure S4, the signal intensity is not significantly affected by other biological interference. Na_3_VO_4_, a commonly used and competitive phosphatase inhibitor (Liu et al., 2017), was then co-incubated with ALP for 30 min before addition of the probe to the test solution. As shown in Supplementary Figure S5, addition of 50 μM Na_3_VO_4_ resulted in a significant reduction in fluorescence intensity. These results confirm that ALP-induced phosphate group-specific cleavage of the probe contributes to the enhancement of fluorescence intensity. Next, the effect of pH on CS-ALP and the enzymatic reaction was also evaluated, with results demonstrating that CS-ALP could perform well at physiological pH (Supplementary Figure S6).

**FIGURE 3:**
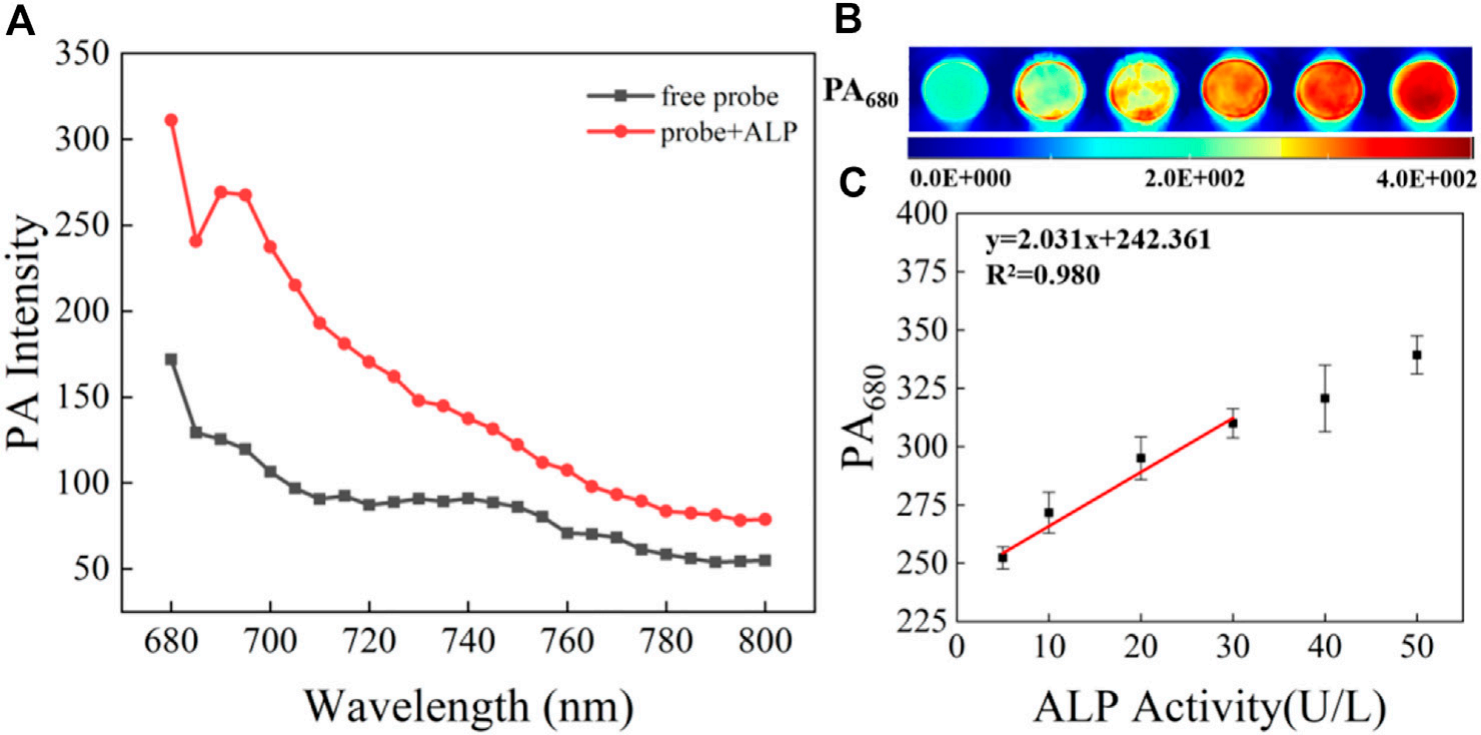
**(A)** PA spectra of 5 μM CS-ALP in the presence or absence of 40 U/L ALP. **(B)** PA_680_ images of CS-ALP (5 μM) in the presence of different concentrations of ALP (0, 10, 20, 30, 40, and 50 U/L). **(C)** Plot of PA_680_ intensity *vs*. ALP concentration.

The detectability of CS-ALP using PA imaging was then tested. We first tested the PA spectra for CS-ALP in the presence of varied ALP activity. The results showed that it was generally consistent with its absorption spectra. As displayed in Figure 3A, the PA signal intensity at 680 nm (PA_680_) exhibited 1.8-fold enhancement at the saturation point. PA_680_ images of CS-ALP were obtained (Figure 3B) by treating the probe with increasing concentrations of ALP (0–30 U/L). The PA_680_ signals displayed linear responses to ALP in the 0–30 U/L concentration range, with a calculated detection limit of 2.5 U/L (Figure 3C).

### Imaging of ALP in the cisplatin-induced AKI cell model

**FIGURE 4:**
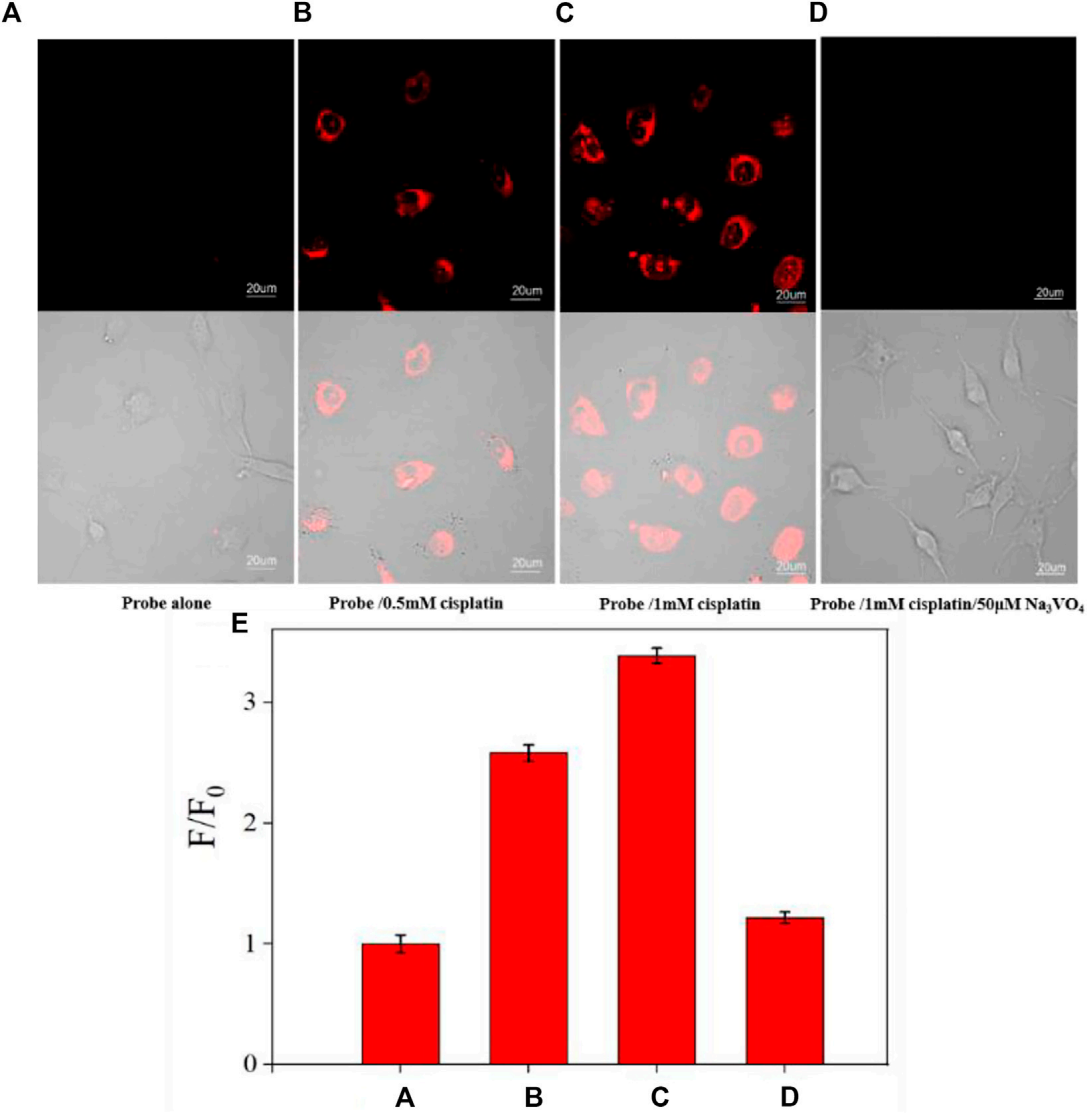
Images of HK-2 cells pretreated with **(A)** 0 mM, **(B)** 0.5 mM, and **(C)** 1 mM cisplatin for 4 h, and then incubated with 5 μM probe for 30 min. **(D)** Images of HK-2 cells incubated with 1 mM cisplatin and 50 μM Na_3_VO_4_ for 4 h, and then incubated with 5 μM CS-ALP for 30 min. **(E)** Normalized average signal intensity in the aforementioned images A–D. *λ*_ex_ = 635 nm, *λ*_em_ = 680−750 nm, scale bar: 20 μm.

The cytotoxicity of CS-ALP was first assessed using the MTS assay. The results showed that high cell viability was maintained after co-incubation with 2–10 μM probe, indicating that CS-ALP had good biocompatibility (Supplementary Figure S7). Next, we used HeLa cells for co-incubation with the probe for live-cell imaging experiments because ALP is overexpressed in HeLa cells. As shown in Supplementary Figure S8, when excited at 635 nm, HeLa cells showed bright fluorescence. However, when pretreated with Na_3_VO_4_ before co-incubation with CS-ALP, HeLa cells showed negligible fluorescence. The results demonstrate that the signal enhancement in HeLa cells is due to ALP activity. We then constructed an AKI cell model using cisplatin, a drug known to induce AKI. HK2 cells treated with CS-ALP alone were excited at 635 nm and showed virtually no fluorescence (Figure 4A). At the same time, we observed that, when pretreating HK2 cells with 0.5 mM or 1 mM cisplatin, signal variations indicated cisplatin dose dependence (Figures 4B,C) and the fluorescence intensity ratio increased by 2.6 and 3.4 times, respectively (Figure 4E). Furthermore, when the cisplatin-treated HK2 cells were co-incubated with Na_3_VO_4_ for 30 min before addition of CS-ALP, there was almost no fluorescence (Figure 4D). These results suggest that CS-ALP could be used for measurement of endogenous ALP in cisplatin-treated HK2 cells, and that the activity of ALP is directly related to the process of cisplatin-induced AKI.

### Detection of ALP in urine and blood samples collected from drug-induced AKI mouse

**FIGURE 5:**
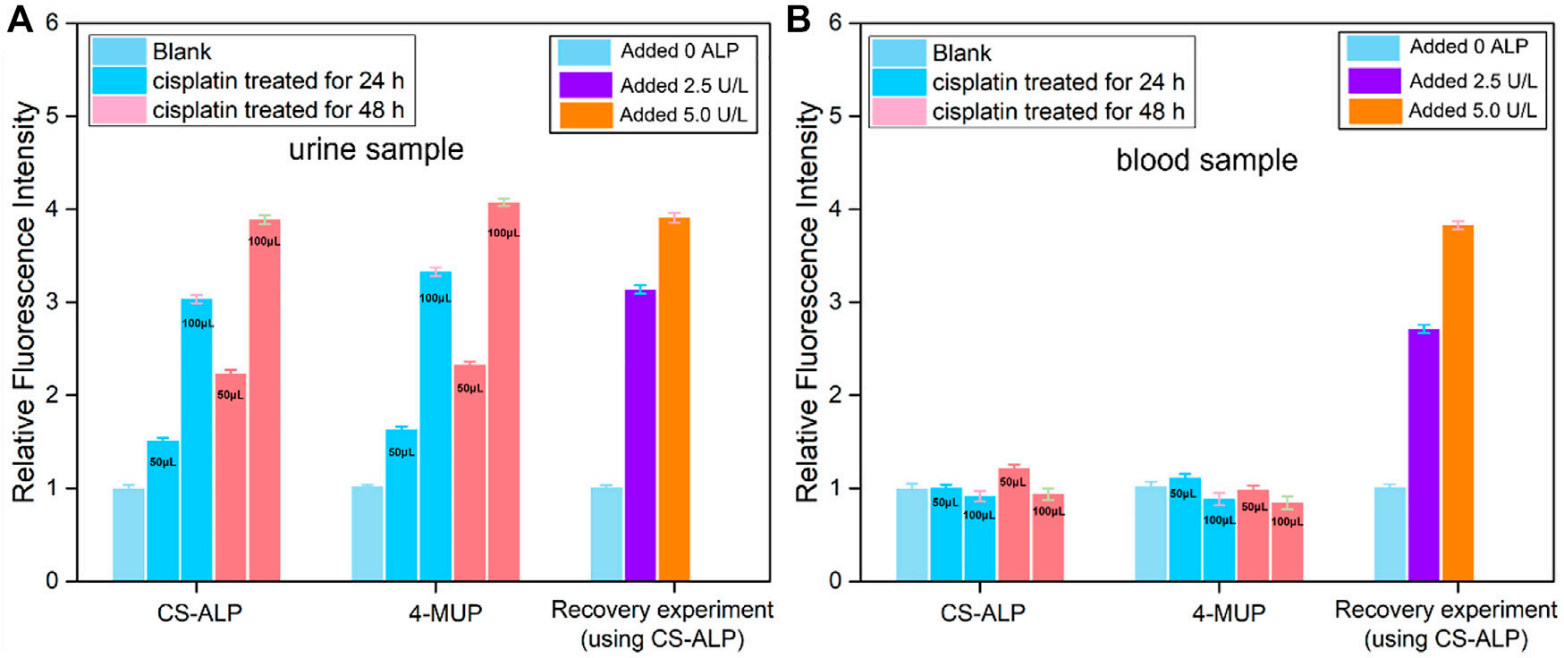
Using CS-ALP/4-MUP to test the ALP activity in urine and blood samples. The fluorescence intensity of CS-ALP/4-MUP at 716 nm and 450 nm in mouse urine samples **(A)** and blood samples **(B)** excreted by healthy and AKI mice. The mouse urine samples were obtained using metabolic cages, and the blood samples were obtained through retro-orbital sampling. The 50 or 100 μL urine or blood samples were immediately added to the test solution (total volume 1 ml). Fluorescence intensity in the healthy mouse was defined as 1.0.

The AKI mouse model was established by intraperitoneal injection of 20 mg/kg cisplatin or normal saline as control. We first tested the urine samples of mice and found that the signal intensity from mice treated with cisplatin for 24 h and 48 h was 1.51-fold and 2.23-fold higher, respectively, than that of the control group. Blood sample analysis showed limited signal increase in the experimental groups compared to control. This result is consistent with that observed when using the commercially available ALP probe 4-MUP (Figure 5). These results are also consistent with a previous study showing that ALP was discharged into the urine following cellular injury due to early stage AKI (Charlton et al., 2014). According to the fluorescence correction curve for CS-ALP to ALP, the calculated ALP activity levels in the urine of AKI mice were 30.1 U/L (cisplatin-treated 24 h) and 45.6 U/L (cisplatin-treated 48 h), respectively. Thus, the CS-ALP probe may be an effective tool for early detection of AKI.

### Bimodal NIRF/PA imaging of ALP in the drug-induced AKI mouse

**FIGURE 6:**
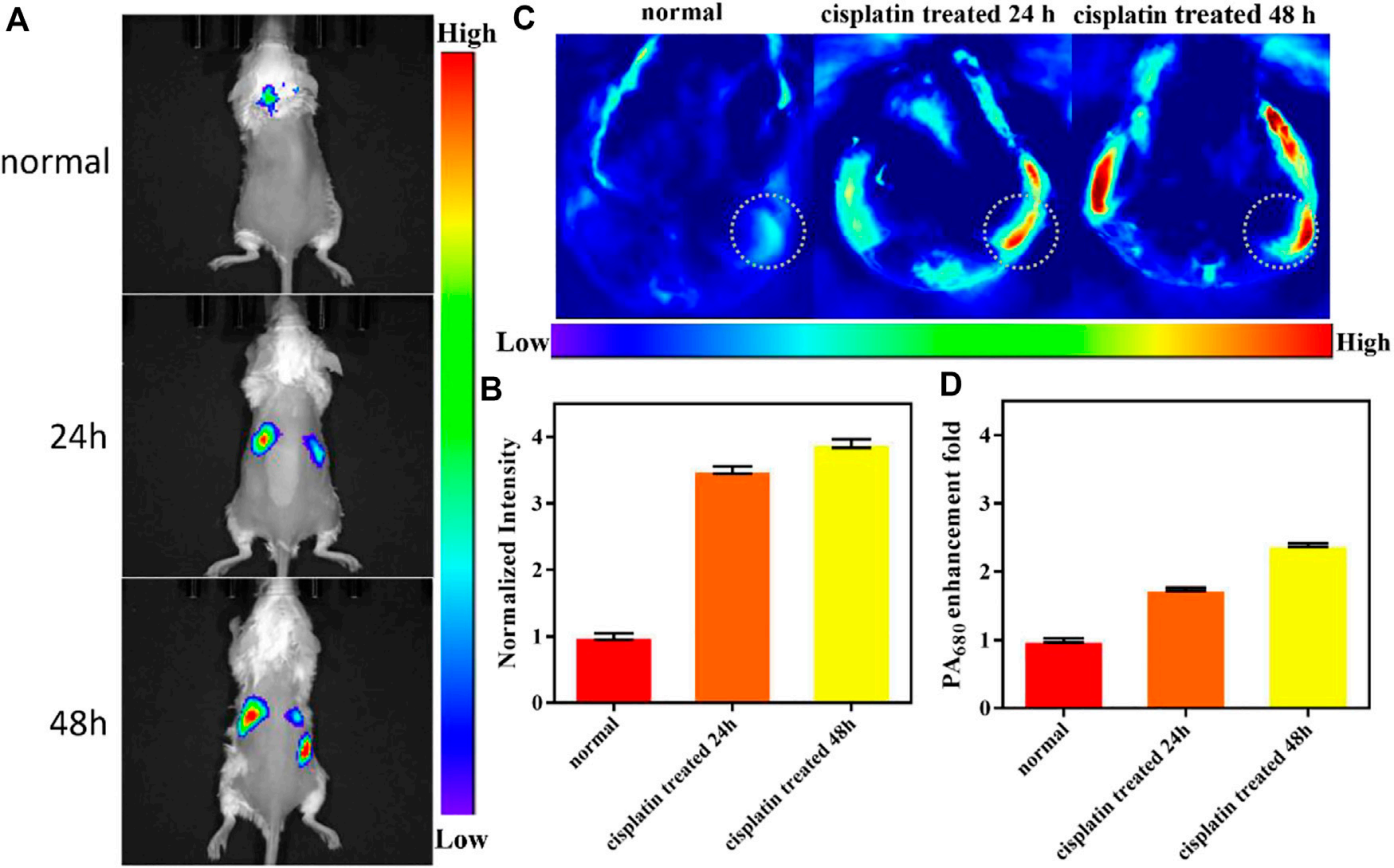
**(A)** *In vivo* imaging of a mouse after treatment with saline or cisplatin (20 mg/kg) for 24 h or 48 h, followed by injection (i.v.) of 0.15 mg/kg CS-ALP (in DMSO/DPBS, 1:4 v/v) for 20 min. Excitation filter 640, emission filter ICG. **(B)** Mean intensity ratio in **(A)**. **(C)** PA_680 nm_ images from the kidneys of mice after pre-injection (i.p.) with saline or cisplatin (20 mg/kg) for 24 h or 48 h, followed by injection (i.v.) of 0.15 mg/kg CS-ALP (in DMSO/DPBS, 4:1 v/v) for 1 h. **(D)** Mean intensity ratio in **(C)**. Values are the mean ± SD, *n* = 3.

After successful NIRF imaging of ALP in the AKI cell model, we applied CS-ALP to *in vivo* bimodal NIRF/PA imaging using commercial *in vivo* imaging systems. First, we constructed an AKI mouse model according to our previous protocol (Liu H.-W. et al., 2020). The biodistribution of CS-ALP after intravenous (i.v.) injection showed that CS-ALP remained inactive in the control mouse but was activated in the AKI model mouse (Supplementary Figure S9A). In another group, imaging of isolated organs of the AKI mouse at 1.5 h post injection showed that CS-ALP accumulated primarily in the kidneys and liver (Supplementary Figure S9B), indicating that cisplatin may induce liver injury and kidney injury at the same time (Palipoch and Punsawad, 2013). As mentioned earlier, the log P value of CS-ALP is 2.238, which indicates that the probe has good water solubility and shows the potential for use in imaging to assess renal metabolism. Subsequently, 0.15 mg/kg CS-ALP was intravenously administered at different cisplatin post-treatment time points (24, 48, and 72 h). Real-time *in vivo* imaging showed that CS-ALP could be rapidly activated in the kidneys at 10 min post injection and that the signal intensity was stable at 10–30 min, followed by an obvious decrease at 120 min post injection (Supplementary Figure S10). Therefore, we timed the *in vivo* NIRF imaging to be at 30 min after injection of the probe. As displayed in Figures 6A,B, the NIRF signal intensity gradually enhanced, mainly in the kidneys of mice treated with cisplatin for 24 h or 48 h. The signal ratios for the 24 h and 48 h groups were 3.52-fold and 3.92-fold higher, respectively, than that of control group, indicating that ALP was upregulated in the kidneys of cisplatin-treated mice. We also carried out PA imaging, and the results are shown in Figure 6C and Supplementary Figure S11. The PA imaging data indicate that the kidney regions of the control group (treated with normal saline) were low contrast at 1 h post injection of CS-ALP, while the PA signal in the kidney regions of cisplatin-treated mice gradually increased. The average signal intensity is displayed in Figure 6D, which indicates that the probe could be used for semi-quantitative analysis of ALP in drug-induced AKI. The PA imaging results were consistent with the NIRF imaging results, but the *in vivo* PA imaging required a longer time to obtain high contrast results compared to NIRF imaging due to the high background signal with PA imaging in this region, hence the advantage of dual-mode imaging for acquisition of more reliable *in vivo* imaging results (Luo et al., 2020; Zhang et al., 2020). Hematoxylin–eosin (H&E) staining revealed normal tubular morphology in the control group, compared to widely spaced tubules, damage to the brush border, and formation of hyaline casts within the damaged tubules in the experimental group 24 h after treatment with cisplatin (Supplementary Figure S12). Considering these results, we conclude that upregulation of ALP expression will occur at the early stages of AKI and will continue to be expressed with the progression of AKI. Additionally, we demonstrated the development and validation of use of CS-ALP in NIRF/PA bimodal imaging as a tool for evaluating drug-induced AKI in response to ALP.

## Conclusion

In summary, we constructed a NIRF/PA bimodal sensor CS-ALP for the detection and imaging of ALP in cisplatin-induced AKI that has high sensitivity and specificity both *in vitro* and *in vivo*. The probe is chemically stable in the presence of ROS at physiological concentrations and allows high-fidelity imaging results. Upon addition and with increasing activity of ALP, the absorption intensity of the probe at 685 nm gradually increased and was accompanied by a NIRF enhancement with a peak at 716 nm, which were used for “turn-on” PA and NIRF imaging, respectively. According to the fluorescence titration experiment, the calculated detection limit was 0.26 U/L. CS-ALP was used in the imaging of ALP in HK2 cells after stimulation by cisplatin. Furthermore, CS-ALP is water-soluble and showed good renal-targeting capability for NIRF/PA bimodal imaging of ALP in a cisplatin-induced AKI mouse model. The results also indicate that upregulated expression of ALP occurs at early stages of AKI and continues with the progression of AKI. Our work in development of the CS-ALP bimodal molecular probe will not only facilitate mechanistic study of the roles of ALP in drug-induced AKI but may also assist in early diagnosis of AKI.

## Data Availability

The original contributions presented in the study are included in the article/Supplementary Material; further inquiries can be directed to the corresponding authors.
