# Supplementary material for: In situ detection of alkaline phosphatase in a cisplatin-induced acute kidney injury model with a fluorescent/photoacoustic bimodal molecular probe
Source: Front Bioeng Biotechnol. 2022 Nov 23;10:1068533. doi: 10.3389/fbioe.2022.1068533 (PMC9727191; doi:10.3389/fbioe.2022.1068533)
Supplement: Supplementary file 1 [file DataSheet1.pdf]

Supplementary Material

In situ detection of alkaline phosphatase in cisplati -induced acute kidney injury model with a fluorescent/photoacoustic bimodal molecular probe

Xingwang Chen[b], Zhiyang Yuwen[b], Yixing Zhao[b], Haixia Li[b]*, Kang Chen[a,b]* and Hongwen Liu [a,b]*

[a] Department of Hepatobiliary Surgery, the First Affiliated Hospital of Hunan Normal University (Hunan Provincial People's Hospital), Hunan Normal University, Changsha, China

[b] Key Laboratory of Chemical Biology and Traditional Chinese Medicine Research, College of Chemistry and Chemical Engineering, Hunan Normal University, Changsha, China

Email: liuhongwen@hnu.edu.cn

contents

Experimental Section.......... S3

Scheme S1.......... S5

Table S1 .... S6

Fig. S1 .... S7

Fig. S2 .... S8

Fig. S3 .... S8

Fig. S4..........S9

Fig. S5..........S9

Fig. S6..........S10

Fig. S7..........S10

Fig. S8..........S11

Fig. S9..........S11

Fig. S10..........S12

Fig. S11..........S12

Fig. S12..........S12

Mass Spectra and ^{1}HNMR S13

References S15

EXPERIMENTAL SECTION

Reagents and Apparatus. Water was purified and doubly distilled by a Milli-Q system (Millipore, USA). Photoluminescent spectra were recorded at room temperature with a HITACHI F7000 fluorescence spectrophotometer (1 cm standard quartz cell) with excitation and emission slit are set at 5.0 nm and 10.0 nm respectively. Mass spectra were performed using an LCQ Advantage ion trap mass spectrometer (Thermo Finnigan). NMR spectra were recorded on a Bruker DRX-400 spectrometer using TMS as an internal standard. All chemical shifts are reported in the standard δ notation of parts per million. Thin layer chromatography (TLC) was conducted using silica gel 60 F254, and column chromatography was carried out over silica gel (200-300 mesh), both of them were obtained from Qingdao Ocean Chemicals (Qingdao, China). The pH was measured with a Mettler-Toledo Delta 320 pH meter. All photoacoustic images were analyzed and collected at various time points by an InVision 256-TF imaging system (iTheraMedical).

ALP was purchased from Sigma-Aldrich, cisplatin was purchased from Chemical Energy. Other chemicals used in the synthesis of probe were purchased from commercial suppliers and used without further purification.

Spectrophotometric Experiments. Both the fluorescence and UV-Vis absorsption measurement experiments were carried out in 10 mM tris buffer saline (TBS, pH 8.0). The fluorescence emission spectra were recorded at an excitation wavelength of 680 nm with emission wavelength ranged from700 to 900 nm. The test solution of the CS-ALP (5 μM) in 2 mL of 10 mM TBS (pH 8.0) was prepared by placing 50 μL of the CS-ALP stock solution (1.0×10^{-4} M) in 2mL of the various analytes buffer/DMSO solution. The resulting solutions were kept at 37 °C for 30 min and then the fluorescence intensities were measured.

Fluorescence microscopy imaging in live cells. Because phosphate could inhibit the activity of ALP, in the cell imaging results, we coincubated the cells with probe in the TBS (pH 7.4, 10 mM) buffer solution. To investigate the capability of probe CS-ALP for detection ALP in living cells, cells were first seeded in a 30 mm glass-bottom dish plated and grown to around 80% confluency for 24 h before the experiment. The cellular experiments can be divided into two groups. The first group is that HeLa cells were incubated with 5 μM CS-ALP for 30 min, then the cells were washed by TBS buffer before imaging. In the second group, after pretreatment with Na_3VO_4 for 4 h, HeLa cells were incubated with 5 μM CS-ALP for 0.5 h, then the cells were washed by TBS prior to imaging.

For the cisplatin-induced nephrotoxicity model in HK-2 cells, the cellular experiment can be divided into four groups. The first group is that the HK-2 cells were treated with 5 μM CS-ALP. The second and third group is that HK-2 cells were pre-treated with different concentrations of cispaltin (500 μM or 1000 μM) for 4 h, respectively, and then treated with 5 μM CS-ALP for 30 min. Then the cells were washed by TBS buffer before imaging. The fourth group, cells were pre-incubated with Na_3VO_4 (50 μM) in the presence of cisplatin (1 mM) for 4 h, and then treated with 5 μM CS-ALP for 30 min, then the cells were washed by DPBS buffer before imaging. Confocal fluorescence imaging of cells was performed using an Olympus FV1000 laser confocal microscopy (Japan).

Cisplatin-induced acute kidney injury. Cisplatin was dissolved in 0.9 % saline. BALB/c mice were intraperitoneally preinjected with cisplatin (20 mg/kg) for 24 h and 48 h, respectively, then intravenously injected with the probe CS-ALP (0.15 mg/kg, in 20% DMSO / DPBS solution). NIRF and PA imaging was observed under an IVIS Lumina XR (IS1241N6071) and InVision 256-TF imaging system (iTheraMedical, Germany) in vivo imaging system respectively.

Histopathological Studies. Renal tissues of BALB/c mice were fixed in 10% formaldehyde immediately following sacrifice, processed for histological examination according to a conventional method, and stained with hematoxylin and eosin (H&E).

Synthesis and Characterization

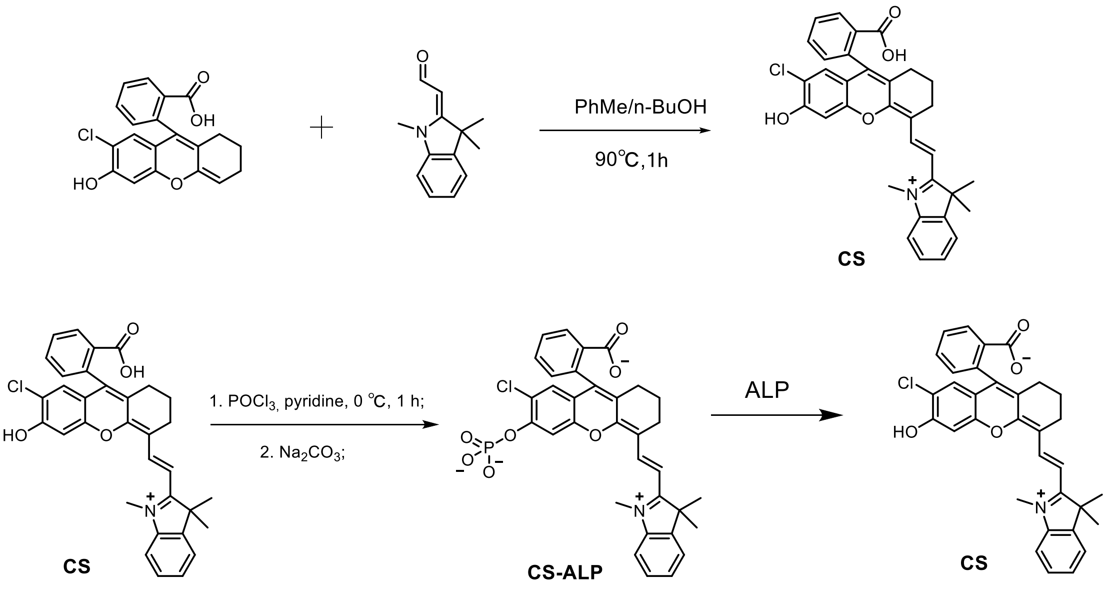

Scheme S1. Synthesis of CS-ALP and the reaction mechanism of CS-ALP with ALP.

Synthesis of CS. CS was prepared by the reported methods.[1] ^{1}HNMR (500 MHz, DMSO-d_6) δ 8.07-8.02 (m, 2H), 7.48 (m, 2H), 7.43-7.41 (d, J = 7.42 Hz, 1H), 7.28-7.26 (t, J = 7.27 Hz, 1H), 7.08-7.06 (d, J = 7.07 Hz, 1H), 7.04-7.00 (m, 2H), 6.72 (s, 1H), 6.33 (s, 1H), 5.75-5.73 (d, J = 5.74 Hz, 1H), 3.36 (s, 3H), 2.58 (m, 2H), 2.28 (m, 1H), 2.12 (m, 1H), 1.68 (s, 6H), 1.24 (m, 2H). MS (EI): m/z, 538.16, $[M]^+$, calcd. 538.18.

Synthesis of CS-ALP. CS (0.54 g, 1 mmol) was dissolved in dry CH_2Cl_2 (20 mL) and stirred at 0℃. And $POCl_3$ (0.2 mL) and pyridine (0.3 mL) were added though syringe. After that, the reaction solution was stirred at room temperature for 2h. Then, ice water (50 mL) was added, and used Na_2CO_3 to adjust the pH to 7. and the reaction solution was extracted with CH_2Cl_2/EtOH. The combined organic phase was dried with Na_2SO_4, and concentrated under reduced pressure. After purified by the silica gel chromatography (CH_2Cl_2/EtOH, 3:1, v/v), compound **CS-ALP** was obtained as a blue solid (0.18 g, 31%). ^{1}HNMR (500 MHz, DMSO-d_6) δ 8.18 (s, 1H), 8.00 (d, J = 8.2 Hz, 2H), 7.69 (s, 1H), 7.67-7.61 (m, 3H), 7.53-7.28 (m, 3H), 6.72 (d, J=16.1 Hz, 2H), 5.34 (s, 2H), 3.14 (s, 3H), 2.92 (m, 2H), 2.06 (m, 2H), 1.78 (s, 6H), 1.48 (m, 2H). MS (EI): m/z, 616.10, $[M]^-$, calcd. 617.14.

probe	$\lambda_{ab}/\lambda_{em}$ (nm)	detection limit	Imaging model	Application	Ref.
HTQPA	410nm/550nm	1.36 mU/mL	Fluorescence imaging	In Situ Localization of ALP in Live Cells	Liu et al., 2017
Y1<br>Y2<br>Y3<br>Y5	421nm/512nm<br>406 nm /532 nm<br>404 nm /481 nm<br>405 nm /522 nm	Not mentioned	Two-Photon Fluorescence Imaging	Organelle-Specific Detection of Phosphatase Activities	Zhou et al., 2016
HBTP-mito	660 nm/ 703 nm	0.72 mU/mL	Ratio metric<br>Fluorescence Imaging	Imaging Alkaline<br>Phosphatase Activity in Mitochondria and Tumor	Zhang et al., 2019a
Cy-OP	736 nm /766 nm<br>516 nm /616 nm	0.16 mU/ mL	Ratio metric Near-Infrared<br>Fluorescence Imaging	Detection and Imaging of Alkaline Phosphatase Activity in Living cells	Zhang et al., 2019b

Cyp	690 nm /738 nm	0.095 mU/mL	Near-Infrared Fluorescence Imaging	The Detection of Endogenous Alkaline Phosphatase Activity in Vivo	Li et al., 2017
LET-3	685nm /730nm (FL.) and 710 nm (PA)	0. 2 mU/mL	Near-Infrared Fluorescence and Photoacoustic Dual-modal Imaging	Imaging of Endogenous Alkaline Phosphatase in Tumor	Gao et al., 2019
XQ-P	720 nm/ 770 nm	0.017U /mL	Near-Infrared Fluorescence Imaging	real-time image ALP activity during the diagnosis and treatment of diabetes in mice	Wang et al., 2021b
This work CS-ALP	680nm/716nm(FL.) 680nm(PA)	0.26 mU/mL	Near-Infrared Fluorescent/Photoacoustic Bimodal Imaging	In situ detection and imaging of ALP in drug-induced acute kidney injury	

Table. S1 Recently reported ALP probe, Ref. 13-21.

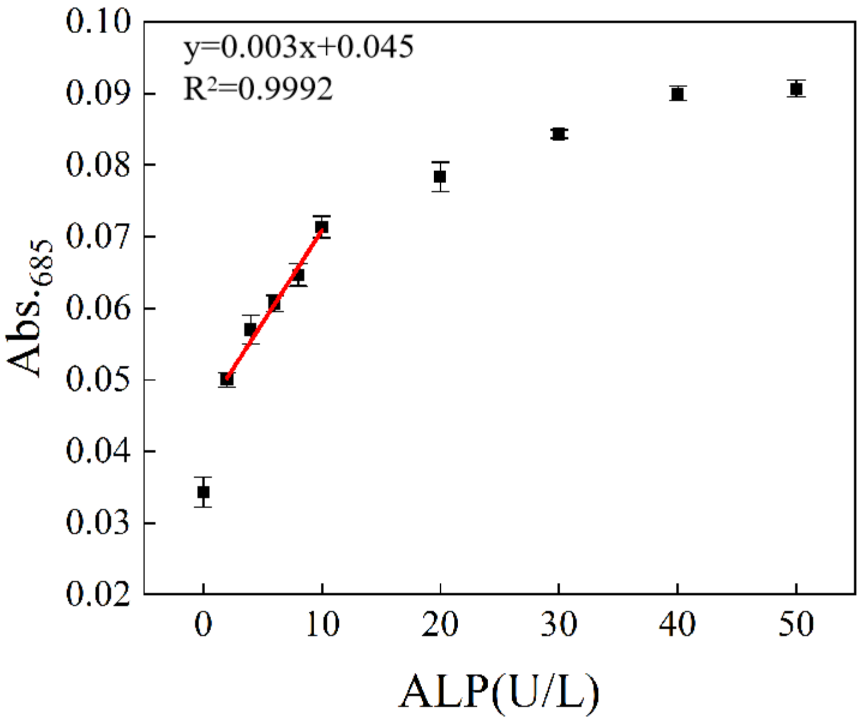

Figure S1 Plot of Abs.$_{685}$ of **CS-ALP** against ALP concentrations and linear relationship in 0-10 U/L ALP range.

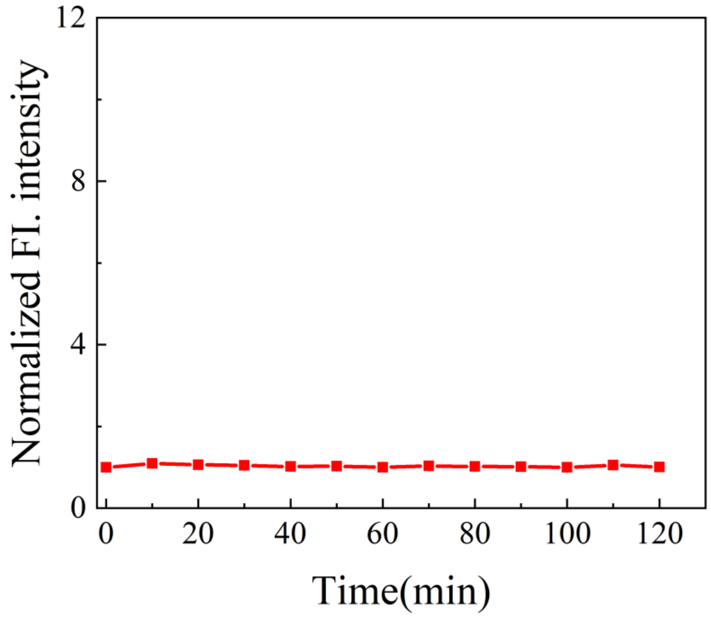

Figure S2 The stability test of **CS-ALP** (5 μM) in TBS solution in the absence of ALP (pH 8.0, 10 mM, 5% DMSO). The results showed that no enhancement of the fluorescence intensity at 713 nm was observed.

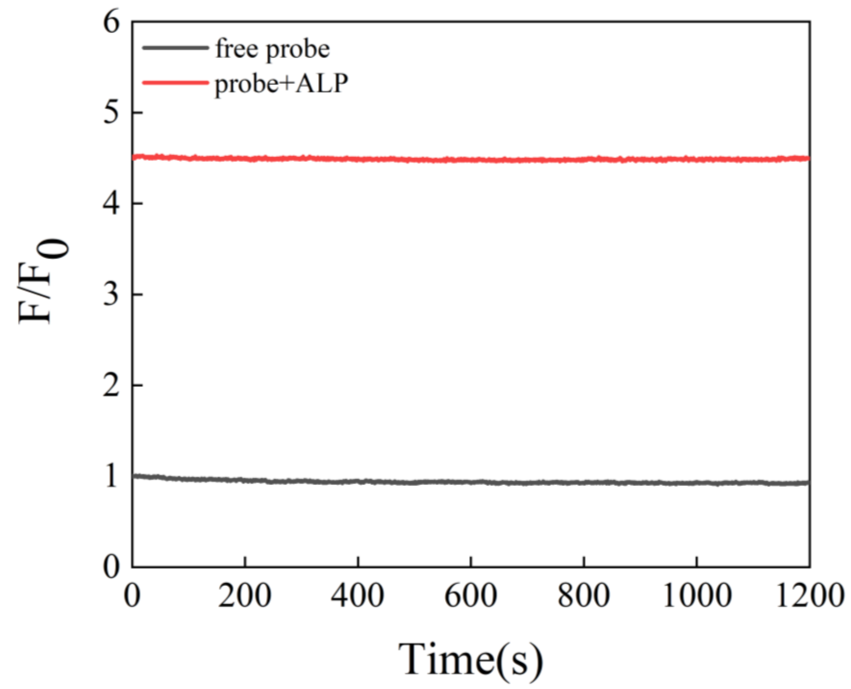

Figure S3 The photo-stability of the probe and reaction product was tested. Recording the fluorescence intensity at 716 nm. E_X Slit: 5.0 nm, E_m Slit: 10.0 nm, PMT Voltage: 900 V.

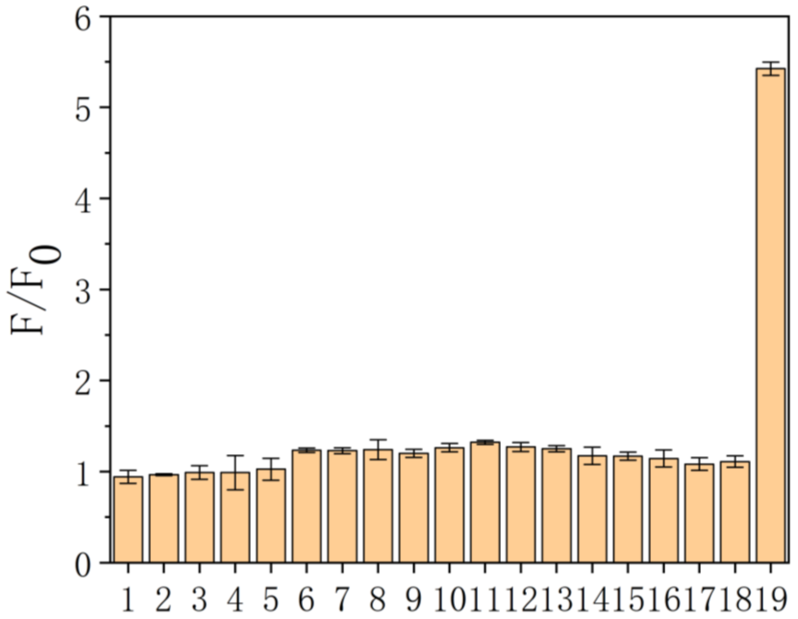

Figure S4 The selectivity test of the probe (5μM) to other substances : (1) blank, (2) 10 μM NaClO, (2) 200 μM H_2O_2, (3) 100 μM $MgCl_2$, (4) 100 μM $CaCl_2$, (5) 100 μM KNO_3 , (6) 100 μM $Ca(NO_3)_2$, (7) 100 μM $ZnCl_2$, (8) 200 μM L-Proline, (9) 200 μM L-Glutamic acid, (10) 200 μM L-Alanine, (11) 200 μM L-Tryptophan, (12) 200 μM L-Methionine, (13) 200 μM L-serine, (14) 200 μM L-Arginine, (15) β-glucosidase, (16) Lipase, (17) trypsin,(18) carboxylesterase, (19) ALP.

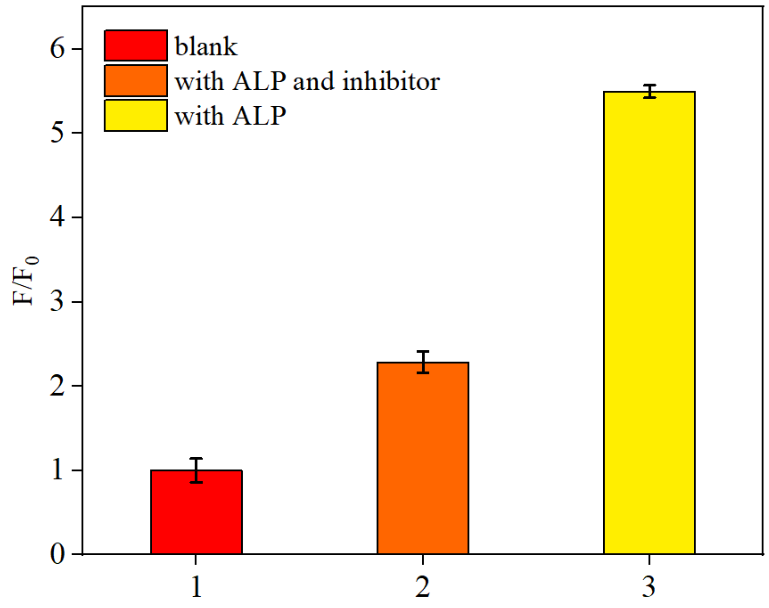

Figure S5 The inhibitor experiment of the probe to ALP inhibitor Na_3VO_4.

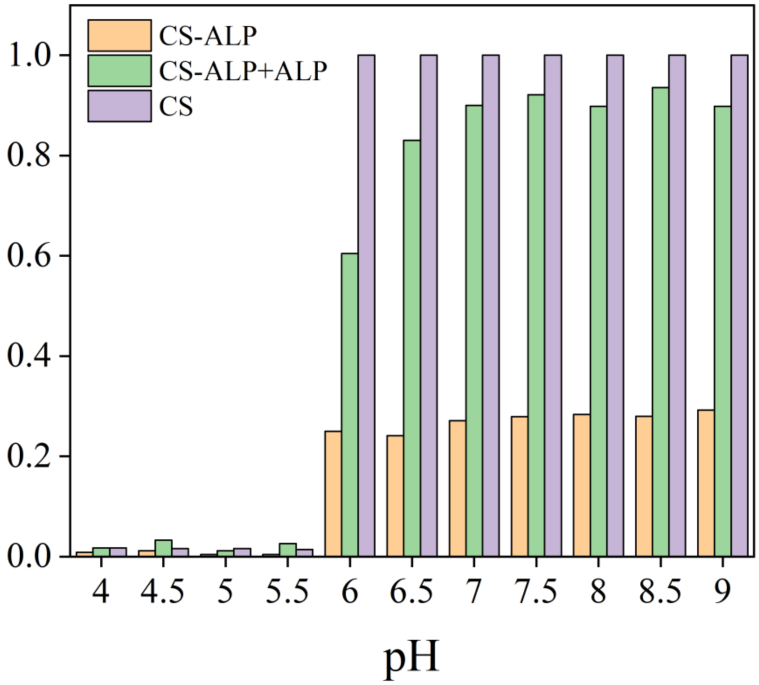

Figure S6 Plot of fluorescence intensity at 716 nm vs pH value. Effect of pH on the fluorescence intensity of 5 μM CS-ALP (yellow column), CS-ALP with ALP (40 U/L) (green column,), and 5 μM CS (purple column). The fluorescence intensity of CS (5 μM) at pH 7.5 is defined as 1. The pH were adjusted by NaOH (aq, 1M) or HCl (aq, 1M), λ_{ex} = 680 nm.

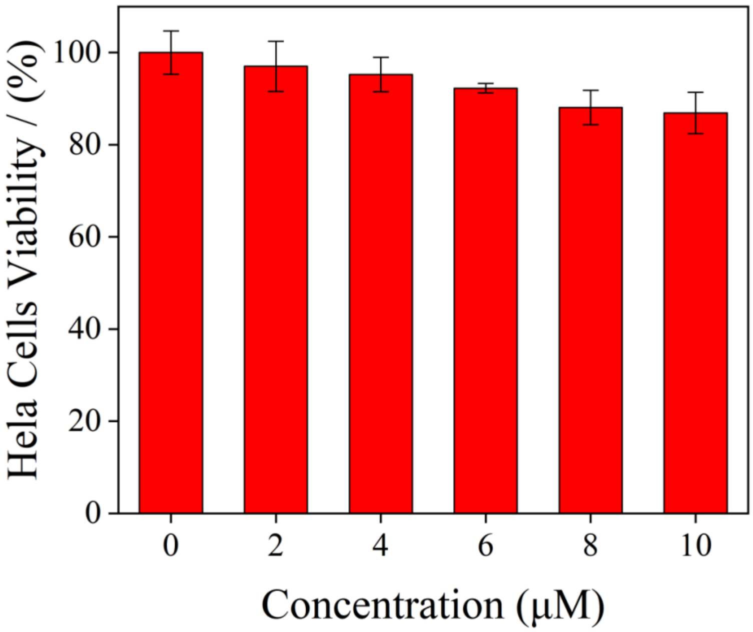

Figure S7 Cytotoxicity of CS-ALP against HeLa cells as determined by MTS assay: HeLa cells were treated with CS-ALP (0-10 μM).

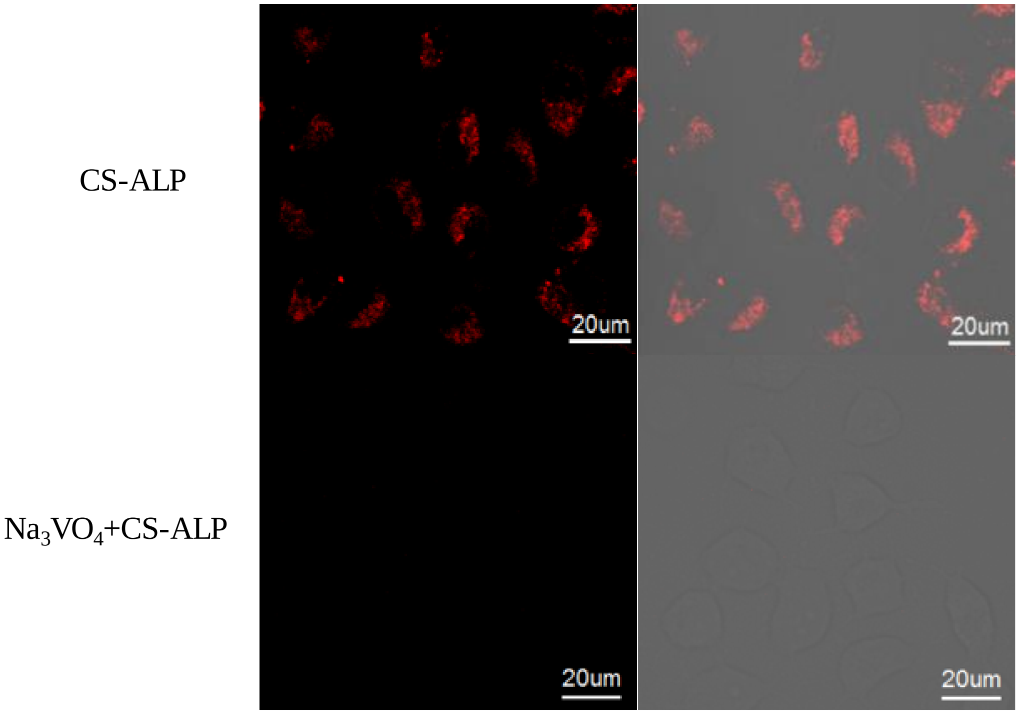

Figure S8 Fluorescence and DIC merge images of HeLa cells. The HeLa cells were incubated with probe for 30 min before imaging or the HeLa cells were treated with Na_3VO_4 for 4 h then incubated with probe for 30 min before imaging. λ_{ex}=635 nm, λ_{em}=680-750 nm, scal bar: 20 μm.

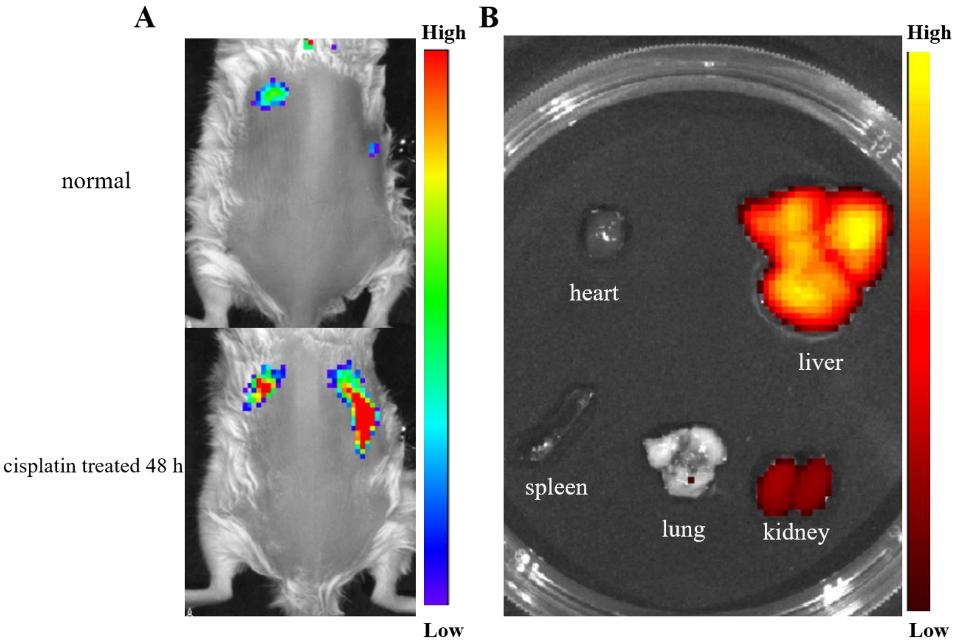

Figure S9 (A) *In vivo* NIRF imaging of mice (normal and cisplatin treated 48 h AKI mouse) after intravenous injection of CS-ALP (0.15 mg/kg) for 1 h. (B) Fluorescence images of the internal organs at 1.5 h post injection of CS-ALP after anatomy. Excitation filter 640, emission filter ICG.

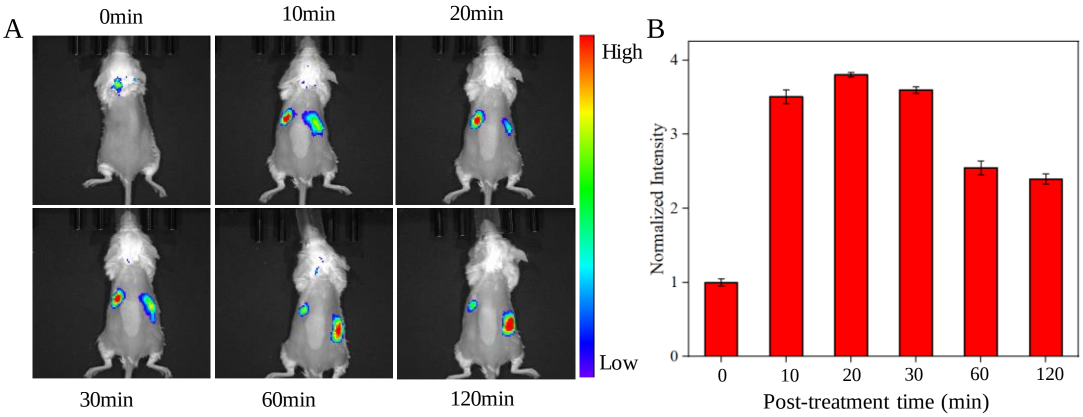

Figure S10 (A) *In vivo* NIRF imaging of cisplatin treated for 24 h mice after intravenous injection of CS-ALP (0.15 mg/kg), the images were obtained at 0, 10, 20, 30, 60 and 120 min. (B) Average intensity ratio in (A).

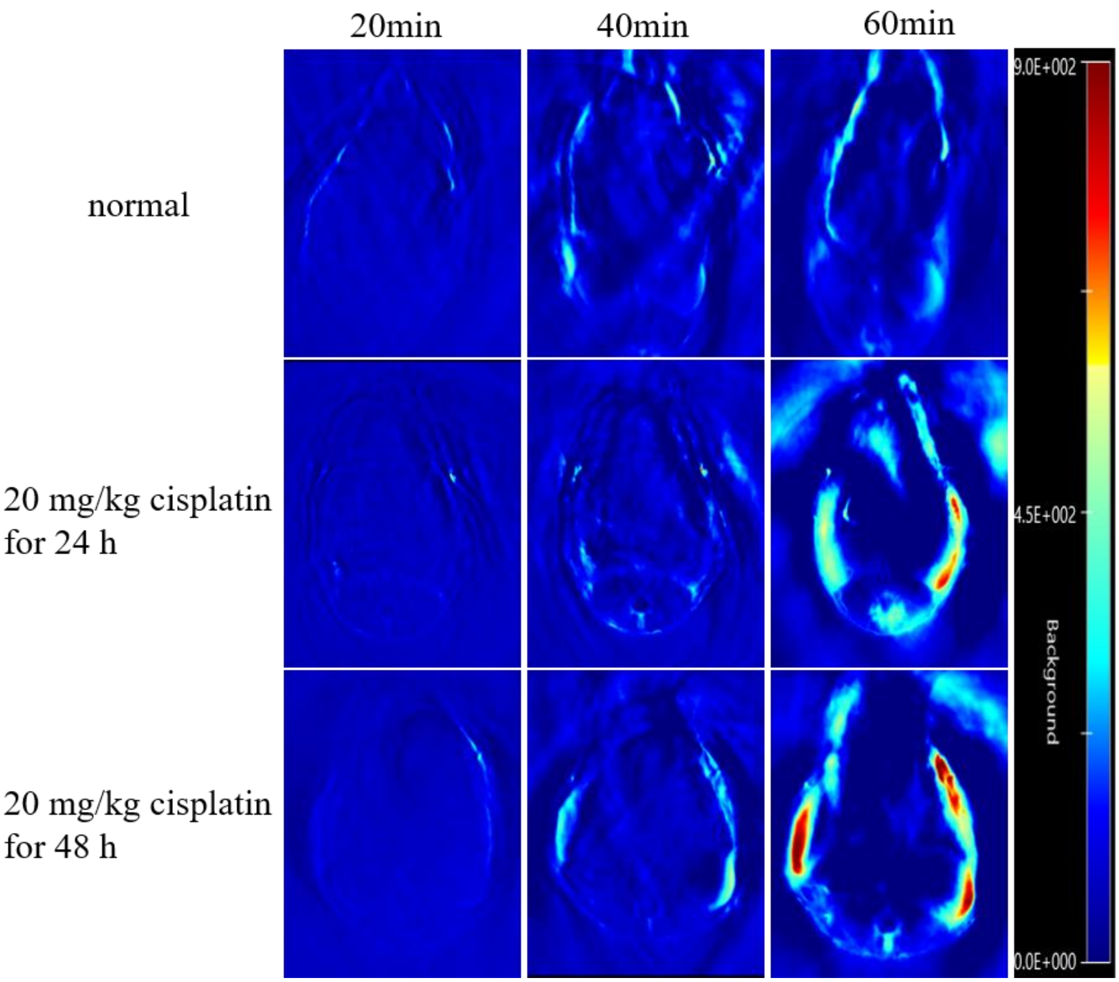

Figure S11 *In vivo* PA_{680} images from the kidneys in living mice after intraperitoneally pre-injected with saline, 20 mg/kg cisplatin for 24 h or 48 h then intravenous injection of CS-ALP (0.15 mg/kg) in for 20 min, 40 min and 60min.

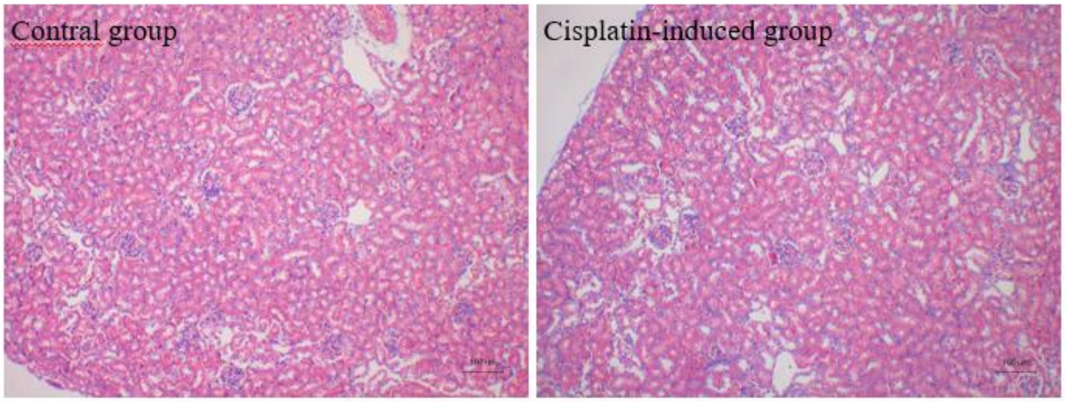

Figure S12 Representative photomicrographs of H&E staining in paraffin-embedded kidney sections from normal mouse or mouse treated with cisplatin for 48 h.

Mass Spectra and ^{1}HNMR

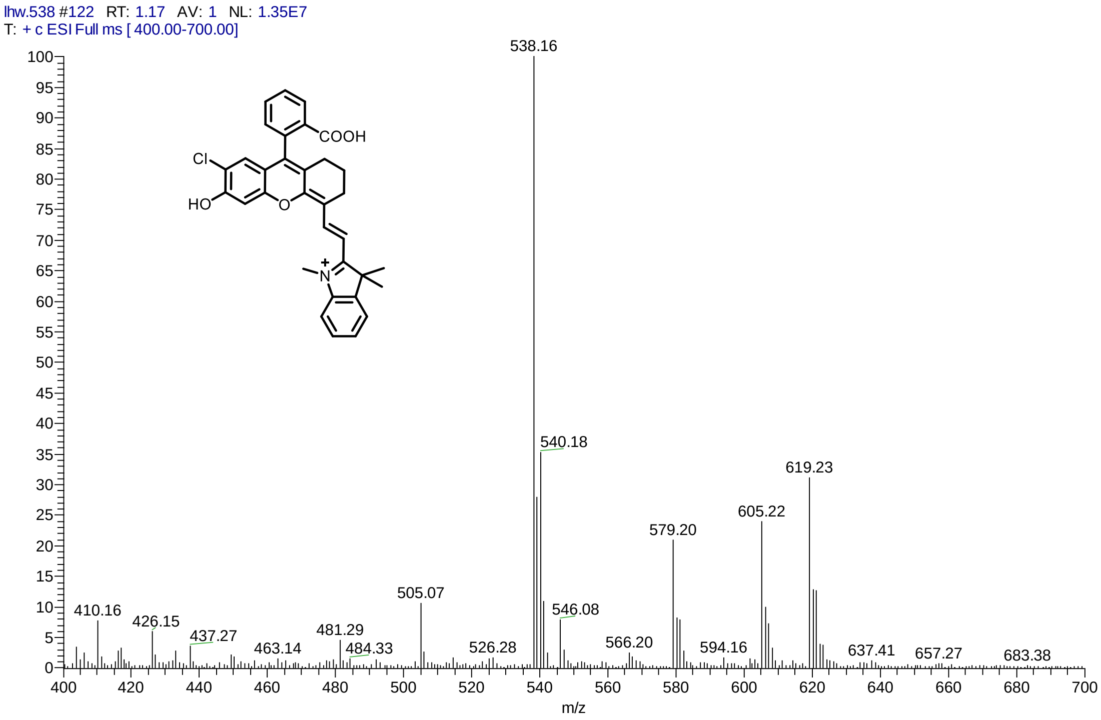

Figure S13. EI mass spectrum of the **CS**.

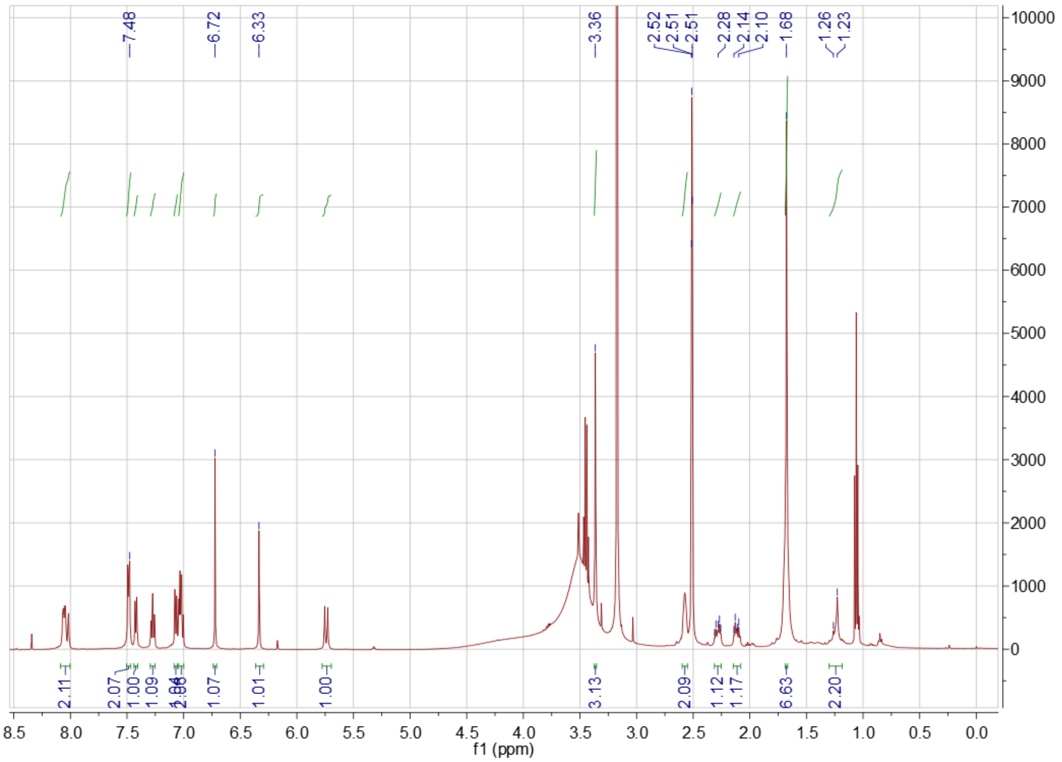

Figure S14 ^{1}HNMR spectrum of the **CS**.

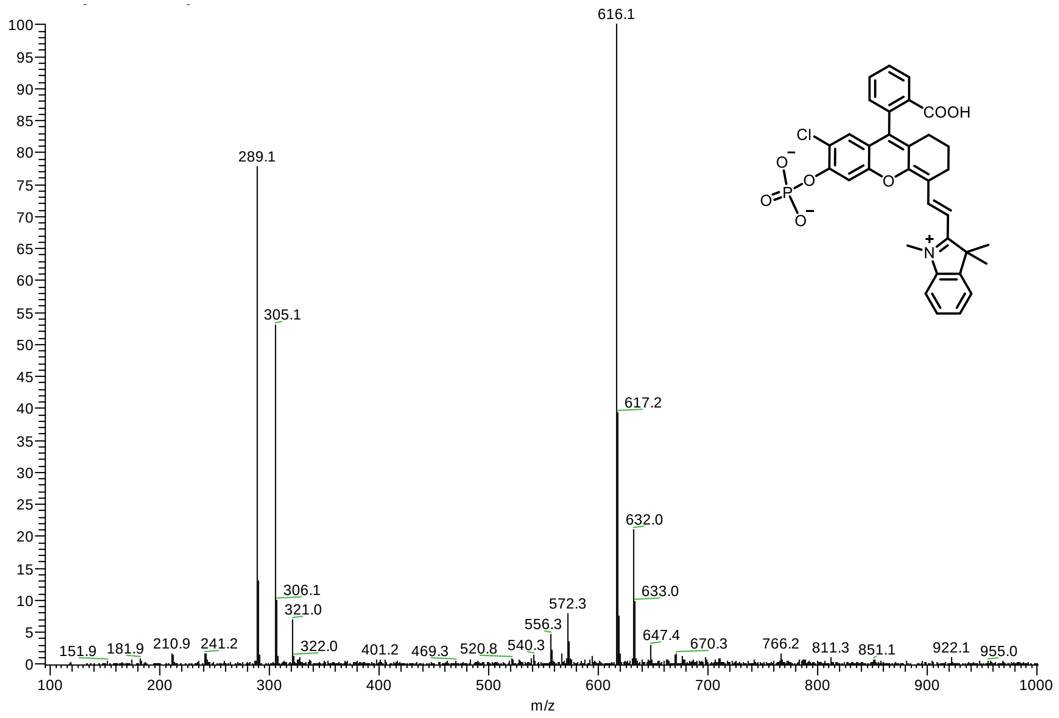

Figure S15 EI mass spectrum of the **CS-ALP**.

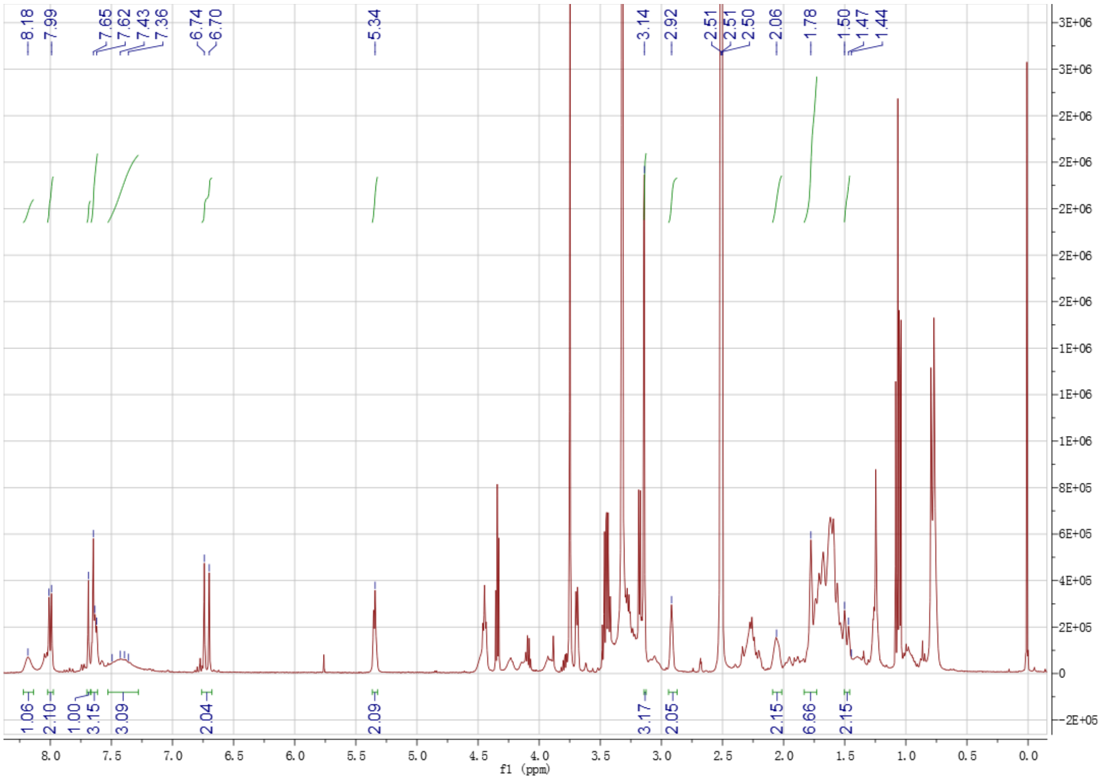

Figure S16 ^{1}HNMR spectrum of the **CS-ALP**.

REFERENCES

(1) Xiong, X.; Song, F.; Chen, G.; Sun, W.; Wang, J.; Gao, P.; Zhang, Y.; Qiao, B.; Li, W.; Sun, S.; Fan, J.; Peng, X. Construction of Long-Wavelength Fluorescein Analogues and Their Application as Fluorescent Probes. *Chem. Eur. J.* **2013**, *19*, (21), 6538-6545.
